# “*So, we started to say hi to each other on campus*.” a qualitative study about well-being among PhD candidates in Norway

**DOI:** 10.1080/17482631.2025.2474355

**Published:** 2025-03-17

**Authors:** Erica Marie Kunz-Skrede, Marianne Molin, Miroslava Tokovska

**Affiliations:** a Department of Health Sciences, School of Health Sciences, Kristiania University of Applied Sciences, Oslo, Norway; b Department of Nursing and Health Promotion, Faculty of Health Sciences, Oslo Metropolitan University, Oslo, Norway

**Keywords:** PhD candidates, well-being, social capital, social belonging, social support

## Abstract

**Background and Purpose:**

Loneliness, social isolation, and lack of social belonging are factors that may negatively impact the mental health and well-being of PhD candidates. This study aims to advance understanding of the function of social activities in their role as interventions that foster social belonging and well-being among PhD candidates.

**Methods:**

After collecting observational data from the well-being interventions, 10 PhD candidates were interviewed to explore how they perceived their participation in social activities on campus and how it affected their sense of social belonging.

**Results:**

Our results show that participating in social activities was beneficial for PhD candidates on both a personal and professional level, potentially leading to an increased sense of community and well-being, along with increased social interaction, networking, and collaboration. PhD candidates’ well-being was found to be linked to social capital in the forms of social belonging and social support.

**Conclusion:**

Organizing social activities tailored to PhD candidates’ needs may help increase their sense of well-being by generating social capital, which could benefit PhD candida nationally and worldwide.

## Introduction

Research on the well-being of PhD candidates in a social capital setting reveals significant concerns. Well-being can be defined as “how people feel and how they function, both on a personal and a social level, and how they evaluate their lives as a whole” (Michaelson et al., 2012, p. 6). It has been observed through various studies that individuals pursuing a PhD often face challenges related to their psychological well-being, displaying higher rates of behavioural disorders, somatic symptoms, anxiety, and depressive tendencies in comparison to those who choose not to continue their education beyond a master’s degree (Holbrook et al., 2024). Moreover, elements such as age, gender, nationality, finance and work, the duration of the PhD studies, and engagement in postgraduate activities are identified as key factors that significantly influence the well-being and mental health status of students (Pizuńska et al., 2021). An increased focus on activities that foster well-being among the general population has shown that participating in activities such as cooking and collective eating might cultivate feelings of meaning, purpose and positive reinforcements (Farmer & Cotter, 2021). Research has also shown that people who eat in a social environment are more likely to have social networks that can provide both social and emotional support (Dunbar, 2017). Furthermore, recent research on potential well-being interventions for PhD candidates has shown that organized self-care workshops might foster well-being by providing arenas where the different stressors experienced during doctoral education can be addressed (Daly & Gardner, 2022).

In higher education the term “social capital” mainly refers to resources embedded in students’ social networks, such as social support; further, it is argued that social belonging is crucial for students’ well-being and success, and it is an important element for generating social capital (Ahn & Davis, 2020). Higher education is increasingly focusing on the mental health and well-being of PhD candidates, and it is evident that various factors, including social isolation, loneliness and lack of social affiliation, can contribute to the development of mental health problems and negative well-being during doctoral education (Evans et al., 2018; Litalien & Guay, 2015; Sverdlik et al., 2018; Zhang et al., 2022). Several studies have investigated the factors contributing to negative mental health and well-being, but there is still a need for more knowledge about possible interventions that foster positive mental health and well-being among PhD candidates (Evans et al., 2018; Sverdlik et al., 2018; Zhang et al., 2022). In 2018, a significant prevalence of individuals with depression and anxiety was found among PhD candidates in 26 countries by Evans et al. (2018). In addition, the study found that graduate students are six times more likely to experience depression and anxiety than those in the general population. Sverdlik et al. (2018) examined the factors affecting the well-being of doctoral students, and their findings suggest that the demanding nature of doctoral programmes often leads to the students’ personal health and social life being neglected. High demands and workload, feeling guilt for taking time away from academic work, social isolation and work-life imbalance were found to be correlated with high levels of burnout, depression, and negative well-being among doctoral students.

Although research on PhD candidates has increased in the last couple of years there is still a need for more, particularly regarding different possible well-being interventions that foster social belonging. It is also argued that research on possible interventions that target social belonging among PhD candidates can help identify social factors that are able to mitigate negative mental health and well-being, along with determining factors that foster positive mental health and well-being (Zhang et al., 2022). This study aims to advance understanding of social activities as possible well-being interventions that foster social belonging among PhD candidates, and is guided by the research question: *How do PhD candidates perceive their participation in social activities on campus, and how does taking part affect their sense of social belonging?*

### The conceptual framework

Social capital theory as a conceptual framework was utilized to advance understanding of proposals for different well-being interventions. Social capital has been understood and approached on different levels of society; the political scientist Robert Putnam understood social capital on a collective level and defined social capital as “features of social life—networks, norms, and trust—that enable participants to act together more efficiently to pursue shared objectives” (Putnam, 1995, p. 665). It is also argued that social capital is generated through repeated face-to-face interaction within both formal and informal social networks (Wollebæk & Selle, 2007), and Putnam (1995) described social capital as a resource that can contribute to bridging social cleavages in society. The sociologist James Coleman also understood social capital on a collective level and argued that certain forms are generated through social structures and social relations within society (Coleman, 1988). The French sociologist Pierre Bourdieu understood social capital on an individual level and investigated the connection between different forms of capital and how they influence human beings and their individual lifestyle choices in everyday life (Giddens & Sutton, 2017). Social capital consists of several different dimensions, and there are different ways to operationalize this theoretical perspective; in this study it is operationalized through the dimensions of social belonging and social support.

Social belonging is defined as feeling safe, at ease, being connected and respected, and is one of several concepts within the social capital theory (Ahn & Davis, 2020). Social capital, particularly collective social capital inspired by Robert Putnam, has been formulated around the components of trust, social network, and participation, and is presumed to contribute to a safe, happy, healthy and efficient society. In addition, social belonging in higher education is described as being linked to students´ personal feelings of being related and connected to their respective institutions, and it is argued that a sense of social belonging is crucial for their well-being and success. Social relations that enhance social belonging are also argued to augment the subjective perception of meaning and quality of life (Lambert et al., 2013). Furthermore, Ahn and Davis (2020, p. 14) argue that the “sense of belonging in higher education and social capital is sustainably related”, and describe social capital, in the form of network and interaction, as playing an important part in students´ sense of social belonging. They also describe a strong overlap between social belonging and social capital and argue that social belonging can be used as a tool to measure social capital.

Social capital theory provides a multifaceted framework for understanding how social connections and networks generate resources that benefit both individuals and communities. While earlier sections focused primarily on bonding social capital through social belonging, the theory encompasses three distinct but interrelated forms: bonding, bridging, and linking capital (Putnam, 2000). Bonding social capital refers to close relationships between similar individuals, such as PhD candidates within the same programme, characterized by strong emotional connections and mutual support. These horizontal ties foster trust, reciprocity, and shared identity—elements that emerged clearly in our findings around increased social belonging. Bridging social capital describes connections between heterogeneous groups or individuals across social divides. For PhD candidates, this manifests in relationships across different departments, disciplines, and cultural backgrounds. Putnam (2000) argues that bridging capital is especially valuable for information diffusion and expanding opportunities, as diverse networks expose individuals to different perspectives and resources. The social activities in our study deliberately facilitated such bridging connections by bringing together candidates from various programmes. Linking social capital represents vertical relationships across power differentials within institutional hierarchies (Claridge, 2018). For PhD candidates, this includes connections with supervisors, senior researchers, and university administration. While our activities primarily targeted peer relationships, the development of informal networks may help candidates better navigate institutional structures and access organizational resources.

These three forms of social capital interact dynamically within higher education settings. Strong bonding capital within peer groups can provide the confidence and support needed to build bridging connections across departments. Similarly, bridging capital may facilitate access to linking capital as candidates expand their professional networks. Understanding these interactions is crucial for designing interventions that holistically support PhD candidates’ social integration and professional development. This expanded theoretical framework guides our analysis of how organized social activities contributed not only to emotional support and belonging (bonding capital), but also to cross-programme collaboration (bridging capital) and institutional integration (linking capital). It also provides insight into how different forms of social capital might buffer against isolation while enhancing both personal well-being and academic success.

Sidney Cobb (1976), p. 300) defines social support as “information leading the subject to believe that he is cared for and loved, esteemed, and a member of a network of mutual obligations”; he notes that social support promotes well-being, and the concept of social support is divided into emotional support, esteem support, instrumental support and informational support, all of which are important across the lifespan. Feeney and Collins (2015) argue that well-functioning and supportive relationships between individuals are fundamental for individual and collective well-being. Small acts, such as words of encouragement, enthusiastic responses to good news, and being mentally and physically present and attuned to others and their needs, can increase personal well-being.

## Methods and methodological considerations

### Study design

A qualitative design with a phenomenological approach and semi-structured in-depth individual interviews were used, to both gain a deeper understanding of the phenomenon of social belonging among PhD candidates and to develop knowledge about participants’ subjective experiences of organized social activities related to social belonging (Green & Thorogood, 2018). In addition, an observational design was used to gain an insider’s perspective on how the PhD candidates interacted with each other (Malterud, 2017); field notes were taken after each activity, according to an observation protocol (Brottveit, 2021).

### Study setting

Two types of organized social activities were chosen for this project—cooking and social eating, and self-care workshops (Appendix 1 – Activity plan). Each activity was conducted twice, with approximately three weeks between each. Both activities were chosen to foster well-being among PhD candidates by generating social belonging and social support. The cooking and social eating activities were arranged on campus to create an environment where PhD candidates from different PhD programmes would collaborate on making food, followed by sharing the meal. The self-care workshops consisted of the theory of the importance of self-care during doctoral education, as well as different self-care and self-management exercises. The self-care workshops aimed to provide tools for PhD candidates to help and support each other during their doctoral education, as well as for practising self-care on their own.

### Participants and recruitment

A total of 31 PhD candidates at one University College in Norway were purposively recruited by the first author via email between August and October 2023. Participants who were employed PhD fellows or admitted PhD candidates during the recruitment period were eligible. PhD candidates who participated in a minimum of one of four activities were invited to participate in interviews concerning the organized social activities. PhD candidates who were not admitted to a PhD programme nor employed as a PhD fellow during the autumn semester of 2023 were excluded from this study.

Fourteen PhD candidates participated in a minimum of one of four organized social activities. During the study, three participants dropped out due to time constraints and high work demand, and one participant did not respond. None of the participants withdrew their consent for observational data to be collected.

### Data collection

A total of four women (*n* = 4) and six men (*n* = 6) participated in the interviews. Three participants were Norwegian, and seven participants were international students from various countries. The interviews followed a semi-structured interview guide (Appendix 1 – Interview guide) and were conducted in English (EMKS); nine interviews were conducted face-to-face on campus, and one interview was conducted online via Zoom. The interviews (*n* = 10) were conducted between 22 January and 2 February 2024 and the average duration was 29 minutes. They were recorded verbatim and were immediately encrypted and stored in a secure Norwegian cloud service according to the General Data Protection Regulations (GDPR). Transcribed interviews were anonymized, with everyone assigned a number, and information that could identify specific individuals was rewritten. This ensured that none of the participants would be recognized in the written results. Additionally, observation protocols (Appendix 3 – Observation protocol) from each of the social activities were followed to supplement the data. Observational data was collected between October 2023 and January 2024. This study followed the COREQ guidelines for qualitative research (Tong et al., 2007). To ensure the validity, reliability, and trustworthiness of the study, the interview guide was pilot-tested by a non-participant not connected to the study, and all study participants were sent the verbatim transcriptions and results for feedback, corrections and approval of the content (Kvale & Brinkmann, 2015; Majid et al., 2017).

The participants were asked topic-relevant questions such as: (1) “How would you describe your experience when participating in the social activities?” (2) “How would you describe your sense of community and belonging among your peers after participating in the social activities? (3) “Describe your understanding of social support and its role in your life”. (4) “How did the social activities you participated in impact your sense of peer support?” (5) “Tell me more about your perception of support among your peers and how it influences you socially and professionally”. The open-ended nature of these questions allowed the participants to explore their personal experiences with the social activities and their impact on social belonging (Green & Thorogood, 2018). To ensure the anonymity of all participants, a combination of letters and numbers was used to report qualitative quotes (e.g., P1, P2, P3).

### Data analysis

Deductive thematic analysis was conducted for the evaluation of the data. This method is suitable for identifying, analysing and reporting themes within data (Braun & Clarke, 2006; Clarke & Braun, 2017), and involves organizing information under specific themes and analysing what is said, rather than how it is said (Brottveit, 2021 (see Table 1). The analytical process followed the following six steps, proposed by Braun and Clarke (2006, p. 87): (1) familiarization with the data through repeated listening to audio recordings and reading of transcriptions (EMKS); (2) generating initial codes and assigning them to identified topics (e.g., friendly relationships, well-being, building of new networks) (EMKS & MT); (3) organizing codes into potential themes (e.g., building social bonds and a sense of community, increasing social interaction and sense of well-being) (all authors); (4) reviewing and validating themes in connection with the conceptual framework (MM & MT); (5) proofreading defined themes to ensure clear categorizations of the narrative (all authors); and (6) analysing and reporting current findings by selecting relevant statements that illustrate the themes (EMKS & MT). Some statements have been edited to improve readability, although care was taken to ensure that all edited statements remain as close to the original statement as possible. The analysis was conducted by three researchers, whose diverse professional backgrounds contributed to the discussion; one researcher has a background in acupuncture and applied public health science (EMKS), the second researcher has an interdisciplinary background within nutrition, epidemiology, and education (MM), and the third researcher has a background in social sciences and public health (MT).

**Table I.** Example of analysis inspired by Braun and Clarke (2006, p. 88).

Data extract	Initial codes	Theme
“I do feel … after doing these sessions and talking more with people outside of my own institution, I am a lot closer to those people. So now we have a much friendlier relationship than we had before”.	Closer relations Friendly relationships	Building social bonds and a sense of community

### Research ethical considerations

This study was approved by the Norwegian Centre for Research Data (Sikt), (Ref. 260541, approved 8 September 2023). All participants were informed about the purpose of the study via email; it was also explained that they could withdraw from the study at any given time without consequences. Informed consent was collected both in writing and orally.

## Results

The results present significant insights from PhD candidates’ perspectives on the impact of social activities on social belonging. The results fall into four main themes: (1) building social bonds and a sense of community; (2) increasing social interaction and sense of well-being; (3) facilitating networking and collaboration through social support; and (4) expanding the range and continuation of social activities.

### Building social bonds and a sense of community

According to most of the PhD candidates, participating in social activities allowed them to establish closer contact and social ties within their PhD programmes. While some already had friendly relationships with their colleagues, others reported that they had formed closer and friendlier relationships after participating, both within and between their programmes. For example, P2 stated:

I participated to (*get to*) know people. And I made many friends, actually … because we were only in contact with the people that we are sitting in a room with.

P4 emphasized:

I do feel… after doing these sessions and talking more with people outside my own institution, I am a lot closer to those people…. now we have a much friendlier relationship than we had before. So, while before you would say hello and be pleasant when you meet them, now it’s more like greeting a friend rather than greeting a colleague!

Although some participants reported experiencing a sense of community within their respective peer groups, others felt that participating in the activities further enhanced their sense of belonging across PhD programmes. This was particularly due to the enjoyable nature of the activities, which encouraged personal relationships between participants. For example, P8 stated:

I would definitely say that my sense of community and belonging is enhanced! The activities created a distinct environment that didn´t involve discussions about research or work. The PhDs (*students*) from different departments were successfully connected, resulting in a larger group.

Also, P9 stressed:

My overall thought is that it’s a good way to connect with people. We have gotten to know each other better, I know people better across (*different*) programmes and everything.

Observations from each activity showed that the participants gradually became friendlier with each other throughout the research, and during the last activity it was noticeable that several PhD candidates had established networks beyond their doctoral studies. The discussions during the activities showed a genuine interest in bonding and connecting on a more personal level.

### Increasing social interaction and sense of well-being

The participants also mentioned that the social activities helped to increase the interaction between themselves. For some of the participants, the activities assisted in creating an environment where seeking interaction with their peers became easier and more natural; P2 stated:

After the activities, I think I’m engaging in interaction more. Just seeing them on the corridor, starting a chat or asking them, “how is your PhD going? What was your topic about? What stage are you?” And also talking about the struggles. “Oh, are you also struggling in this? Yeah, me too … ” Now we’re not best friends, but we’re friends. So, yeah, the interactions changed from (*those of*) colleagues to (*those between*) friends, actually! We talk and we are constantly searching for some opportunity to go out again. So, the activities were helpful.

Although some participants emphasized that they already had some social interaction within their respective group of peers, others stated that participating in the activities developed a new need for social interaction with their peers; P5 explained:

In the beginning, it was a bit difficult to be open and straightforward. But then the time we had in the second cooking activity was enough to be more talkative and open and discuss … the initiative to do the activities again maybe shows something … shows that we need to take part in those activities … because the PhD work is a very lonely process and that’s a good opportunity to interact with other people. So maybe a new need emerged after that to repeat these activities together.

Several participants expressed that doctoral education can be a very lonely process, and having both formal and informal arenas for social interaction can help foster happiness and well-being; P4 commented:

Having someone to talk to and just give ideas to back and forth really invigorates my thought process. So, I think it makes me smarter, kind of… it also makes me happier, because doing this PhD on my lonesome would be very depressing and very, very isolated …

P1 stressed:

People having closer interactions (*and*) caring more about your colleagues … the work progression … (*and*) general well-being due to these kind of programmes … I’m super positive (*about*) what has been done and I think it’s going to be really beneficial in the long term.

Next, P10 emphasized:

As long as it’s very informal, I think it affects me very nicely because I’m very social and this meets me as a professional person. I thrive better when I’m happy. I thrive better and I achieve more things. And … I think that it’s good when it’s holistic and not like an extra thing that feels intimidating for those that actually don’t want to be doing lots of social things …

Observations from the final activity also showed that the interaction between the participants was more natural compared to the first activity. It seemed that they had got to know each other on a more personal level, as well as across PhD programmes. The participants appeared to be at ease and took pleasure participating in the activities, highlighting the importance of providing social platforms that enable authentic interactions and promote well-being for PhD candidates.

### Facilitating networking and collaboration through social support

Participating in the social activities also facilitated networking and collaboration between the participants. Some of the participants argued that having social arenas specifically for PhD candidates was beneficial due to their being equal in the academic hierarchy and provided a safe place to talk about their achievements and struggles. For example, P9 stated:

I feel more collective across programmes (*i.e. part of a bigger collective*), I would say. It’s easy to come in as a new student, I think … and feel like people are in a different process. But then these social activities are arenas where everyone is equal … and everyone is at the same stage.

P1 also emphasized:

Having the ability to talk about things without the fear of being kind of judged for your shortcomings, or having some issues that you’re struggling with affecting how, for example, a supervisor would look at you … having peers that you fully trust, and are understanding and empathetic … that is kind of a lifesaver, both in good times and bad times!

Several participants also mentioned how the activities helped them feel more collaborative across PhD programmes. This was mainly due to participants experiencing that asking for help or providing assistance during the activities was easy and natural, which translated into their work life; P3 gave the following example:

The activity, it was like organised in a way that everyone can contribute … and I felt that if somebody is helping in such a social event, you feel more comfortable in asking for help when (*it is*) related to your PhD or related to something else … and now I will feel more comfortable asking about help related to my project than I would have before.

While some participants had already collaborated with their peers, others stated that engagement in the activities proved advantageous for their professional development and provided a platform for networking and collaboration across PhD programmes. This was mostly due to the participants experiencing the same challenges, sense of support, and having common subjects to discuss; P5 stated:

Maybe you start to talk to other people that you wouldn’t do that before this, right? And also, with your colleagues, then you have something in common to discuss. And then this common thing … starting to discuss about this … you can start to discuss about other things as well, right?! So it’s nice to have something in common with colleagues. And I think it helps for professionals also …

Next, P4 stressed:

I would say, looking from a PhD point of view, having your co-workers actually be a part of your project in a positive way does give a lot of positive results. Also, it allows me to take a load off because I can ask my peers questions, so I don’t have to rely solely on my own brain and my own capabilities.

Observations showed that the participants were more collaborative in the final activity compared to the first activity. They were more open to discussing challenges and were more supportive of each other. Additionally, some participants learned that they were at a similar stage within their respective programmes and deliberated on the possibility of collaborating to advance their projects, despite being enrolled on different PhD programmes.

### Expanding the range and continuation of social activities

All of the participants emphasized the importance of expanding the range and continuation of the social activities; this was due to the activities focusing solely on PhD candidates and being fun and non-demanding, as well as being an appropriate arena for networking. P2 aptly summarized the general feeling within the group:

Socially, the institution needs a lot more activities that are specifically for PhD candidates … There should be several reasons for PhD students to hang out with each other, or with (*students from*) other institutions!

P3 added:

I would say that this was a very great initiative … I would never have thought that being socially active, because I was never socially active, can (*reduce your*) stress, can make you (*feel*) a bit more comfortable with different peers … (*the following day after an activity*) I was able to work more effectively.

While the majority of participants argued that the greatest benefits would be provided in social environments where academic work is not the primary focus, some expressed a desire for additional social activities that could enhance both professional networking and social interaction. P7 made several suggestions:

I think it would be nice to have more arenas where you have people presenting their research … and see how seniors present their research, how they communicate with each other, how they can discuss stuff, etc … and then also have the PhD students watching this … creating an arena where people can exchange knowledge. Then I think combine it with social events as well.

Some of the participants also mentioned that activities outside campus would be a good addition to the activities arranged on campus; both P2 and P4 stated:

It could be good if we also had a social gathering outside campus in your project … types of activities … to do with food and drinks … where we do something other than what we are always doing (*on campus*) … taking us out of the (*familiar*) environment would be really good!

The participants also argued that incorporating organized social activities into the programmes, or facilitating the continuation through the participants themselves, would be beneficial for both present and future PhD candidates; P4 explained why:

The initial social events where you encourage people to talk to someone new or interact with someone new, break the ice with someone new … I do feel like that’s very important for PhD students, just because we can be very much in our own heads. So just to force us to actually say hello to a human person who we’re not usually talking to, that would probably be a good thing to incorporate into all PhD programmes!

Next, P9 emphasized:

… mostly that it is very important to have these types of gatherings. And to foster that kind of arena for talking about things outside what we are working on … talk more about how it feels to work with these things. I think it is very valuable.

Also, P8 commented:

We appreciated the cooking session so much that we decided that we want to continue them on our own!

Observations further indicate that the activities were in a setting distinct from their usual work environment, highlighting the potential advantages of expanding the range and continuation of social diversions for prospective and current PhD candidates. Additionally, providing social platforms specifically for PhD candidates seemed to inspire the participants to continue the activities themselves.

## Discussion

This study contributes to existing literature by developing and advancing understanding of the function of social activities in their role as interventions that foster social belonging and well-being among PhD candidates. The four main themes that emerged from the analysis of the collected data through interviews and observations are: (1) building social bonds and a sense of community; (2) increasing social interaction and a sense of well-being; (3) facilitating networking and collaboration through social support; and (4) expanding the range and continuation of social activities.

While organized social activities might generate social capital in the forms of social belonging and social support, there is still a need for more research on possible well-being interventions that might mitigate negative PhD well-being. It is evident that high demands and workload, feeling guilt for taking time away from academic work, social isolation, and work-life imbalance are correlated with high levels of burnout, depression and negative well-being among doctoral students (Sverdlik et al., 2018). Although the sample was small in our study, participation in organized social activities was found to be an important factor to enable PhD candidates to build stronger social bonds and a sense of community, both within their respective groups of peers and across other PhD programmes. This is emphasized due to most participants expressing their opinion that the social activities helped increase their sense of social belonging and community, and the development of closer and friendlier relationships across PhD programmes. The increased sense of belonging and community translated beyond the activities, and one can argue that the development of stronger social bonds, friendlier relationships, and connectedness gained through participating in social activities could be a route to counteracting the feeling of loneliness among PhD candidates. This is supported by findings from Ahn and Davis (2020), who argue that feeling connected to your institution and peers is linked to the development of social belonging.

Participating in social diversions and being encouraged to interact with peers from other programmes also extended beyond the study setting, and the participants started to interact more with each other after taking part in the cooking and social eating activity. Several students participated due to the enjoyable nature of the activities; others took part to enable them to get to know their peers, since they did not have much social interaction aside from the contact with their closest colleagues and supervisors. As the results show, some of the participants started to seek out social interaction with their peers when they previously had not. Additionally, participating in social activities developed a need for social interaction within some students which had been absent before our study began. Although not all participants expressed a change in their interaction pattern, the results indicate that participating in social activities can contribute to increased social interaction among PhD candidates, which is argued to play an important part in PhD candidates´ sense of belonging (Ahn & Davis, 2020).

Furthermore, the results indicate that engaging in social activities and having the chance to interact with similar peers in a relaxed environment enables participants to discuss their work, challenges, and accomplishments, thus contributing to an overall sense of well-being. Even though this study was restricted to four organized activities over four months, the results indicate that organized social activities over a limited period can contribute to the development of social belonging and an increased sense of well-being. These findings are supported by Bye et al. (2020), who argue that the development of social capital is significantly affected by factors such as social interaction and time, and that even small changes over a few weeks are important outcomes. Since doctoral education has experienced an increased prevalence of individuals with depression and anxiety (Evans et al., 2018), and developing social capital takes time (Bye et al., 2020), it is important to further investigate possible interventions that, over time, might mitigate this negative trend.

Participating in organized social activities facilitated networking and collaboration across PhD programmes, and some of the participants emphasized the importance of having social arenas where PhD candidates experience being equal and having the opportunity to network with each other without fear of being judged. However, findings from one study investigating graduate perceptions of doctoral value suggest that the supervisory relationship is of key importance during doctoral education; the same study also argues that the students’ perception of value was influenced by the contact, networks and connectedness they received through support from their peers (Bryan & Guccione, 2018). This supports the notion that social platforms promoting equality are crucial for PhD candidates during their academic journey, as these platforms facilitate the formation of supportive peer connections. Moreover, our study indicates that coordinated social events could enhance collaboration, not only within specific peer groups but also across various PhD programmes. While our data is limited, it can be posited that structuring activities in a manner that encourages seamless collaboration may alleviate difficulties in seeking assistance and solidarity among PhD candidates. Additional findings put forth the notion that students who involve peers in the process of their PhD could yield favourable outcomes in the academic realm. This proposition finds reinforcement in the works of Putnam (1995) and Ahn and Davis (2020), who contend that elements of social life, including networks, have the potential to enhance collective efficiency among PhD candidates.

The dual role of PhD candidates as both students and employees creates a complex professional landscape that can affect their well-being and social capital development (Claridge, 2018). While our study focused on structured social activities, these interventions exist within broader departmental and research group environments that shape candidates’ daily experiences. PhD candidates typically navigate multiple organizational contexts simultaneously—their immediate research group, department, and broader institutional structure. These existing work environments already provide important sources of social capital: research groups offer direct professional support and mentoring; departmental seminars and meetings create opportunities for knowledge exchange; shared office spaces facilitate informal daily interactions; and teaching responsibilities connect candidates with broader academic networks. Our study suggests that social interventions are most effective when they complement these existing structures rather than operating in isolation. For example, participants reported that relationships built during social activities made them more confident contributors in peer group discussions, as well as discussions with supervisors. This highlights how bonding capital developed through informal activities can enhance engagement with formal work structures. However, work environments alone may not fully address candidates’ social and emotional needs, particularly for international students or those in smaller research groups. Strategic social interventions can fill these gaps while building on the professional foundations existing departmental structures provide.

Lack of work-life balance, heightened levels of stress, and elevated expectations for academic output throughout the doctoral journey have all been identified as factors that may have a detrimental impact on the well-being of PhD candidates (Evans et al., 2018; Zhang et al., 2022). The results of our study indicate that broadening the scope and continuity of social engagements could play a significant role in supporting the well-being of PhD candidates. Specifically, engaging in structured social activities was found to potentially serve as a mitigating factor for stress among PhD candidates. The observations made by participants, highlighting that active participation and establishing connections with fellow PhD candidates helped alleviate some of the stress associated with their academic work, lend further credence to this notion. These insights are further supported by Cobb (1976), who posits that the presence of a supportive social network can aid in mitigating environmental stressors and empowering individuals to effectively navigate challenging and demanding circumstances.

Organizing social events that offer PhD candidates a reprieve from their usual work settings could potentially serve as an intervention to foster a sense of social inclusion and well-being. Testimonials from participants underscore the value of being removed from their routine work environment, allowing them some valuable time to step back from the constant pressure to perform. While not specifically addressed in the present study, previous research has explored the benefits of engaging in walking discussions for promoting positive mental health and well-being. These studies have indicated that engaging in conversations while walking in natural settings can enhance overall well-being, satisfaction with work-life balance, and reduce stress-related concerns (Marselle et al., 2013; Van Den Berg & Beute, 2021). Findings from both studies suggest that walk-and-talk should be investigated as a possible intervention that fosters well-being and positive mental health among PhD candidates. Networking and connecting with fellow peers are especially important during the first year of doctoral education, but due to the high academic demands personal health and social life are often neglected areas (Sverdlik et al., 2018). The suggestion of incorporating social activities, as an arena for both social and professional networking and to mitigate negative mental health and well-being, is supported by participant statements indicating that a lack of a supportive peer network can lead to loneliness and lack of motivation during doctoral education.

Our findings suggest that organized social activities serve as a valuable complement to existing institutional support structures for PhD candidates. While departments and research groups provide formal academic networks and mentoring, the informal nature of these interventions created unique opportunities for developing different forms of social capital. The cooking sessions and self-care workshops operated alongside—rather than replacing—traditional academic support systems, filling a distinct social need that formal structures often cannot address. Participants reported that these activities enhanced their ability to utilize existing institutional resources. For example, the confidence and connections built through informal peer interactions made some candidates more comfortable seeking help from supervisors and engaging in departmental seminars. This suggests a synergistic relationship where social capital generated through informal activities strengthens candidates’ engagement with formal support structures. The cross-departmental nature of these interventions also complemented the typically discipline-specific focus of research groups. While maintaining strong ties within their primary academic units, candidates developed valuable bridging capital across programmes—connections that might not naturally emerge through existing structures. This broader network contributed to both personal well-being and professional development opportunities that extended beyond departmental boundaries. While research has documented challenges in doctoral education, our findings reveal a more nuanced picture that includes significant positive experiences. Many participants emphasized the intellectual fulfilment and professional growth inherent in their PhD journey.

The existing departmental structures often provide valuable support and opportunities for development. Candidates reported benefits from engaging in research groups, teaching experiences, and academic discussions. These positive aspects of PhD life complement rather than conflict with the need for additional social support. Understanding this balance helps frame social interventions not as remedies for systemic problems, but as enhancements to an already meaningful academic experience. This more balanced perspective suggests that while social support initiatives are valuable, they build upon existing strengths within doctoral education rather than addressing universal deficits. Future research might explore how positive experiences in PhD programmes can be leveraged to further enhance candidate well-being and success.

### Strengths and limitations of the study

There exist multiple strengths and limitations within this research endeavour. For instance, utilizing available facilities on campus enabled participation, while also enhancing the cost-effectiveness of the study and providing a safe place for the participants. Additionally, the coordination of structured activities, specifically tailored for PhD candidates, enhanced social engagement throughout and following their participation. Despite the strengths observed in this study, limitations are also evident. For example, constraints encompassed the restricted duration of the study and the small sample size. The inclusion of demographic data, and a broad discussion of data saturation, would enhance the reliability and validity of the collected data and facilitate the comparison of the impact of PhD candidates with minority background on well-being during social activities. Moreover, a number of participants solely engaged in the culinary task, thereby yielding insufficient data for the self-care session. Furthermore, it is crucial to acknowledge that both the activities and interviews were administered by the first researcher, potentially leading to response bias. Although the outcomes of this study are not universally applicable to all doctoral candidates, they offer valuable insights into potential well-being interventions warranting further exploration in subsequent studies. Such endeavours should prioritize exploring interventions that nurture enduring social connectedness, a facet not explored in the present study. Moreover, future investigations should delve into how heightened feelings of social connectedness and support can bolster resilience and well-being among PhD candidates. The discoveries stemming from such inquiries could furnish empirical evidence of the enduring impacts of such interventions as structured social engagements. Research in this vein would be well placed to proffer recommendations on interventions that ought to be integrated into doctoral programmes, on both national and international scales.

## Conclusion

While PhD candidates face documented challenges around isolation and well-being, our findings highlight how existing institutional structures and targeted social interventions can work together to foster success. The established academic frameworks within departments and research groups provide essential professional support, which can be enhanced through structured social activities that develop multiple forms of social capital. Our study demonstrates that PhD candidates actively build both bonding capital within their programmes and bridging capital across disciplines when given appropriate opportunities.

The generation of social capital through organized activities complements existing institutional strengths, creating a more comprehensive support system. By recognizing both the challenges and resilience within doctoral education, institutions can strategically implement interventions that build upon current successful practices. This balanced approach—maintaining strong departmental support while fostering broader social connections—may help create an environment where PhD candidates can thrive both personally and professionally.

Future initiatives should focus on integrating social support mechanisms with existing academic structures rather than treating well-being interventions as separate from or remedial to traditional doctoral education. This integration can help institutions leverage their strengths while addressing areas where additional support may be beneficial.

## Appendix 1: Interview guide

**Interview guide**

**“*So, we started to say hi to each other on campus*.” A qualitative study about well-being among PhD candidates in a social capital setting in Norway**

Before the interview:

- Welcome the participant and thank them for participating.
- Short presentation about myself.
- Give information on privacy regulations.
- Ask the participants if anything is unclear and if they have any questions.
- Give information about the purpose of the study and for conducting interviews.

The main purpose of this master´s project is to develop knowledge and a deeper understanding of how organized social activities, such as cooking and eating together, and talking and reflecting on self-care in a social setting, can contribute to creating social belonging and an increased perception of social support among PhD candidates. The research question is: *How do PhD candidates experience participating in social activities and their impact on social belonging?*

- Receive the participant’s consent to the treatment of collected data and to audio-record the interviews (everything that is said, noted and recorded today will be anonymous and treated confidentially).
- I will now start the recording, and everything that is said will now be audio-recorded.

Starting interview questions:

- Tell me something about yourself and your study programme.
- What year of the programme are you in?
- How many of the social activities have you participated in?
- What was your motivation for participating in the project?

Interview questions regarding participating in **organized social activities**

- How would you describe your experience participating in the social activities?

Interview questions regarding **social belonging**

- How would you describe your sense of community and belonging among your peers after participating in the social activities?
- Tell me more about how you experienced the interaction between yourself and the other participants during the activities.
- Describe in more detail the interaction between the PhD candidates after participating in the social activities.

Interview questions regarding social support

- Describe your understanding of social support and its role in your life.
- How did the social activities you participated in impact your sense of peer support?
- Did participation impact your sense of both received and provided support?
- Tell me more about your perception of support among your peers and how that influences you socially and professionally.

Concluding questions

- What changes and improvements would you suggest to enhance the overall social experience for PhD candidates in the future?
- Can you give me any examples of social activities?
- Would you like to add anything about your experience with the social activities that we have not covered in the previous questions?

Closing the interview:

- Thank you for participating in the social activities and the interview.

## Appendix 2: Observation protocol

**Observation Protocol**

**“*So, we started to say hi to each other on campus*.”**

**A qualitative study about well-being among PhD candidates in a social capital setting in Norway**

**Table B1.** Activity 1 – self-care workshop.

Activity 1: Self-care workshop	
Observation question:	Notes:
Who were participating?	
What was the setting?	
What were the participants doing? Which activity?	
How did the participants interact during this activity? - How did the conversations go? - Did they reflect openly with the other participants? - How did the discussions go?	
Any other observations from the activity?	

Own Elaboration, 2024.

**Table B2.** Activity 2 – cooking and social eating.

Activity 2: Cooking and social eating	
Observation question:	Notes:
Who were participating?	
What was the setting?	
What were the participants doing? Which activity?	
How did the participants interact during this activity? - How did the conversations go? - Did they reflect openly with the other participants? - How did the discussions go?	
Any other observations from the activity?	

Own Elaboration, 2024.

**Table B3.** Activity 3 – self-care workshop.

Activity 3: Self-care workshop	
Observation question:	Notes:
Who were participating?	
What was the setting?	
What were the participants doing? Which activity?	
How did the participants interact during this activity? - How did the conversations go? - Did they reflect openly with the other participants? - How did the discussions go?	
Any other observations from the activity?	

Own Elaboration, 2024.

**Table B4.** Activity 4 – cooking and social eating.

Activity 4: Cooking and social eating	
Observation question:	Notes:
Who were participating?	
What was the setting?	
What were the participants doing? Which activity?	
How did the participants interact during this activity? - How did the conversations go? - Did they reflect openly with the other participants? - How did the discussions go?	
Any other observations from the activity?	

Own Elaboration, 2024.

**Table A1.** Activity plan for self-care workshops.

Theme	Self-care workshops
Date and time	23.10.2023 and 04.12.2023
Selection	PhD candidates at one University College in Norway
Goal	Increase awareness of the importance of individual and collective self-care, enhancing social belonging and support, and a sense of well-being.
Actors	Erica Marie Kunz-Skrede, first author May Kunz, Assistant Professor at Inland University of Applied Science (lecturing about self-care)
Actions/preparations	Before the activities • Planning the dates, duration and content for the self-care workshops with the lecturer. • Invite all eligible PhD candidates • Planning the location, room-booking. • Printing out all materials for individual exercises.
	After the activities Evaluate the first activity and receive feedback from participants. Make adjustments.
Materials	Work sheets for individual self-care exercises.
Finances	None
Duration of activities	2 hours per workshop
Duration of preparation and activities	4 hours total for the activity, and 5 hours total preparation
Field notes	Taking fieldnotes after concluding each activity

Activity plan was inspired by Baggers (2009).

Reference: Baggers, J. (2009). Håndbog i aktiviteter. Munksgaard Danmark. 115 p. ISBN 9,788,762,809,413.

**Table A2.** Activity plan for the cooking and social eating activity.

Theme	Cooking and social eating
Date and time	13.11.2023 and 19.01.2024
Selection	PhD candidates at one University College in Norway
Goal	Create an arena for PhD candidates to collaborate and network, enhancing social belonging and support, and a sense of well-being.
Actors	Erica Marie Kunz-Skrede, first author
Actions/preparations	**Before the activities** • Planning the dates, duration, and recipes and cooking instructions. • Booking the teaching kitchen on campus. • Invite all eligible PhD candidates. • Calculate the amount of ingredients
	**After the activities**
	Evaluate the first activity and receive feedback from participants. Make adjustments.
Materials	**Materials at cooking stations (available in the kitchen)** • Chopping boards • Knives • Kettles • Frying pans • Other cooking utensils Spices and condiments (salt, pepper, sugar, oil etc). **Materials for the recipes** • Vegetable soup with sausages: • Carrots • Cabbage • Potatoes
Finances	Approximately 100USD/90EUR
Duration of activities	3 hours
Duration of preparation and activities	6 hours total for the activities and total 4 hours preparation.
Field notes	Taking field notes after each activity.

Activity plan was inspired by Baggers (2009).

Reference: Baggers, J. (2009). Håndbog i aktiviteter. Munksgaard Danmark. 115 p. ISBN 9,788,762,809,413.
